# Characteristics and Microbiological Profile of Patients with Diabetic Foot Infections in Kuantan, Pahang

**DOI:** 10.5704/MOJ.2203.003

**Published:** 2022-03

**Authors:** RY Kow, CL Low, MAS Ayeop, A Che-Ahmad, MS Awang

**Affiliations:** 1 Department of Orthopaedics, Traumatology and Rehabilitation, International Islamic University Malaysia, Kuantan, Malaysia; 2 Department of Radiology, Sultan Ahmad Shah Medical Centre @IIUM, Kuantan, Malaysia

**Keywords:** diabetic foot infection, diabetic ulcer, antibiotic, microbiology, Malaysia

## Abstract

**Introduction:**

The number of people suffering from diabetic foot infection (DFI) has increased precipitously over the years in Malaysia, owing to increased population, urbanisation, the surge of number of people with obesity and physical inactivity. As one of the most dreaded complications of diabetes mellitus, DFI is associated with high morbidity and mortality. We aim to study the microbiological profile of patients with DFI at a university hospital in Kuantan, Pahang.

**Materials and methods:**

This retrospective study was carried out at at Sultan Ahmad Shah Medical Centre @IIUM (SASMEC @IIUM) from 1 January 2018 to 30 April 2019. Patients’ demographic data, types of infection and surgical intervention, and the microbiological profile were obtained from the medical records.

**Results:**

A total of 142 causative pathogens were cultured from 130 tissue samples, with an average of 1.09 pathogens per lesion. Majority of the pathogens were gram-negative pathogens (52.8%). Staphylococcus sp. was the most common pathogen isolated (22.5%). This was followed by Streptococcus sp. (10.6%), *Pseudomonas sp.* (9.2%), *Morganella sp.* (5.6%), *Klebsiella sp.* (4.9%), Enterobacter sp. (4.9%), and others. Among the 142 pathogens, there were 9 multidrug-resistant strains observed. Most of the antibiotics were effective against the gram-positive pathogens except benzylpenicillin, tetracyclin, fusidic acid and ciprofloxacin. Meanwhile, cefotaxime, amoxicillin and ampicillin-sulbactam were also not suitable against gramnegative pathogens. Oxacillin and sulfamethoxazole/ trimethoprim can be used as empirical antibiotics against gram-positive pathogens, while vancomycin should be reserved for patients with septic shock or suspected multidrug resistant strain infection. Piperacillin/tazobactam and ceftazidime can be used as empirical antibiotics against gram-negative pathogens.

**Conclusion:**

Early initiation of empirical antibiotic(s) is paramount to stymie the infection from getting worse while waiting for the identification of causative pathogens in the management of DFI. This study provides a guide for treating physicians to initiate the most appropriate empirical antibiotic in DFI.

## Introduction

Diabetes mellitus, a major non-communicable disease in the world, has imposed a significant burden on the health care system^1^. In the US, financial burden of diabetes mellitus is estimated to cost $327 billion ($237 billion from direct medical cost and $90 billion from reduced productivity)^1^. Adding insult to that, the number of people with diabetes will continue to rise, owing to increased population, urbanisation, the surge of number of people with obesity and physical inactivity^2^. In 2004, Wild *et al* predicted the prevalence of diabetes will be 4.4% in 2030 with a total of 366 million people living with diabetes^2^. In Malaysia, the prevalence of diabetes mellitus has far exceeded the estimation by Wild *et al.* Based on the first National Health and Morbidity Survey (NHMS) in 1986 and the subsequent NHMSs, the prevalence of diabetes in Malaysia has shown a steadfast increment, from 6.3% in 1986 to 17.5% in 2015 (8.3% -1996; 11.6% - 2006; 15.2% - 2011)^3,4^.

Diabetes mellitus is associated with a number of macro- and microvascular complications such as nephropathy, retinopathy, ischemic heart disease, cerebrovascular disease and diabetic foot ulcer^5^. In Malaysia, National Diabetes Registry reports that 1.2% of the diabetic patients have diabetic foot ulcers and 0.9% of them have previous amputations^6^. This is lower than the estimated 15-25% of diabetic patients having diabetic foot ulcers and 4.3% of diabetic patients having had lower leg amputation, possibly due to recruitment of patients only from Health Clinics (Klinik Kesihatan) by the Registry and not from hospitals where most of the patients present with more severe complications^7-10^. In fact, up to 30% of diabetics will develop a foot ulcer, and there is an average of one leg amputation every 20 seconds, causing diabetic foot infection being ranked as one of the top ten diseases that pose heavy burden globally^11-14^.

For the treatment of diabetic foot infection (DFI), aside from surgical procedures, it is paramount to initiate empirical antibiotic(s) early prior to isolation of the causative pathogens, to stymie the infection from getting worse^15^. The empirical antibiotics should be broad-spectrum and target majority of the predicted pathogens in DFI, based on the local microbiological profile^15^. Previous study by Kow *et al* demonstrated that the bacteriology profile of patients in Southeast Asia countries differs starkly compared to other developed countries which show a predominance of grampositive pathogens^5,16^. Even within Malaysia, the microbiological profile of patients with DFI varies among centres, in terms of percentage of gram-staining pathogens and the percentage of culture with growth^5^. With this context in mind, it is important to obtain the local microbiological profile in DFI. We, therefore, aim to describe the characteristics and microbiological profile of patients with DFI at a newly established university hospital in Kuantan, Pahang.

## Materials and Methods

This retrospective study was carried out at at Sultan Ahmad Shah Medical Centre @IIUM (SASMEC @IIUM), a 350bed teaching hospital in Kuantan, Pahang. This study was part of the SANDI project (Septic Arthritis and Diabeticrelated Infection) initiated by the authors. Ethical approval was obtained from the Kulliyah of Medicine Research Committee (Ref: IIUM/305/20/4/1/7). Medical records of patients who were admitted from 1 January 2018 to 30 April 2019 to IIUM MC were reviewed for suitability of recruitment into the study. All patients with diabetic foot infections who had received surgical interventions were included. For patients with history of multiple admissions, only the first admission was included. Patients with incomplete data were excluded from the study.

Demographic data such as age, gender, precipitating factors, duration of illness prior to hospital presentation, types of infection, and types of surgical intervention were extracted from patients’ medical records. The precipitating factors included scratches, insect bite, improper shoe wear, burn, and trauma to the foot such as stepping on a nail or an object falling onto the foot etc. Types of infection were broadly classified into four categories, namely: abscess, infected wound, necrotising fasciitis and gangrene (both wet and dry gangrene), similar as the previous study^17^. On the other hand, types of surgical procedure were classified based on the level of amputation (if amputation was performed). There were also wound debridement, ray amputation or disarticulation of toe, mid- or hindfoot amputation, below-knee amputation, and above-knee amputation. There was no through-knee amputation or hip disarticulation during the study period.

The microbiological profile of DFI was also retrieved from the medical records, including type of specimen, causative pathogens, and the sensitivity of the pathogens to antibiotics tested. Only deep tissue or bone samples obtained during the surgery were included. In accordance with other studies, the samples obtained were incubated at the hospital microbiology laboratory for two days at 37^o^C in blood agar, MacConkey agar and Chocolate agar^5,18^. Conventional method was performed to identify the cultured pathogen and the antibiotic susceptibility testing was carried out based on the National Committee for Clinical Laboratory Standards (CLSI) using the disk diffusion method on Mueller-Hinton agar plates^19^. Likewise, types and dose of antibiotics in the susceptibility testing were similar to previous studies^5,18^. Descriptive data was used for the presentation of results.

## Results

Among the 130 patients included in this study, majority of them were males (n=77, 59.2%), with a male-to-female ratio of 1.45:1 (Table I). Majority of patients were of the elderly age group, with a mean of 62 years (range 31 to 82 years). Most of the patients (67.7%) did not have any precipitating factors prior to developing diabetic foot infections and they normally presented to the hospital within 2 weeks after the development of initial symptoms, with a mean of 11.7 days (range 1 to 90 days) (Table I).

**Table I:** Description of the demographic data of patients included in this study

Factors	Number	Percentage
Gender		
Male	77	59.2
Female	53	40.8
Age^a^ (years)	Mean 62.14	SD 9.952
Duration of illness prior to presentationa (days)	Mean 11.70	SD 10.882
Precipitating factors		
Yes	42	32.3
No	88	67.7
Type of Surgery		
Wound debridement	73	56.2
Ray amputation or disarticulation of toe	33	25.4
Mid- or hindfoot amputation	3	2.3
Below-knee amputation	18	13.8
Above-knee amputation	3	2.3
Type of infection		
Abscess	31	23.8
Infected wound	48	36.9
Necrotizing fasciitis	28	21.5
Gangrene	23	17.7
Haemoglobin (g/dL)^a^	Mean 11.018	SD 2.139
White cell count (x109/L)^a^	Mean 17.58	SD 7.345
Blood cultures (59 samples)		
No growth	52	88.1
Pathogens cultured	7	11.9
Total	59	100
Tissue cultures (130 samples)		
No growth	34	26.2
Monomicrobial	69	53.1
Polymicrobial	27	20.8

^a^Continuous data presented in mean and standard deviation (SD)

More than one-third of the patients were diagnosed to have infected wound (n=48, 36.9%). This was followed by abscess (n=31, 23.8%), necrotising fasciitis (n=28, 21.5%) and gangrene (n=23, 17.7%). Only 16.1% of the patients underwent major amputations (amputations proximal to the hindfoot) as the primary mode of treatment for the DFI. More than half of the patients had wound debridement (n=73, 56.2%), followed by ray amputation or disarticulation of toe (n=33, 25.4%), below-knee amputation (n=18, 13.8%), mid- or hindfoot amputation (n=3, 2.3%) and aboveknee amputation (n=3, 2.3%). In terms of biochemical testing, patients normally presented with leukocystosis with a mean total white cell count of 17.58 x109/L (range 4.7 to 56.3, SD 7.34) and mean haemoglobin level of 11.02 g/dL (range 4.9 to 17.1, SD 2.14).

Out of the 59 blood cultures and sensitivity tests, only 7 yielded cultures. Meanwhile, from the 130 tissue cultures obtained, majority (n=69, 53.1%) yielded single pathogen, followed by no growth (n=34, 26.2%) and polymicrobial (n=27, 20.8%). There were a total of 142 causative pathogens cultured from 130 samples, with a ratio of 1.09 pathogens per lesion. Gram-negative microorganisms outnumbered the gram-positive ones by 5.6% (52.8% versus 47.2%) (Table II). *Staphylococcus sp.* was the most common pathogen cultured (n=32, 22.5%). This was followed by *Streptococcus sp.* (n=15, 10.6%), *Pseudomonas sp.* (n=13, 9.2%), *Morganella sp.* (n=8, 5.6), *Klebsiella sp.* (n=7, 4.9%), *Enterobacter sp.* (n=7, 4.9%), and others (Table II).

**Table II:** Cultured microorganisms in this study

Microorganisms	Number	Percentage
Gram-positive organisms	67	47.2
*Staphylococcus sp*	32	22.5
*Streptococcus sp*	15	10.6
*Enterococcus sp*	2	1.4
*Gamella sp*	1	0.7
Other gram-positive	17	12.0
Gram-negative organisms	75	52.8
*Pseudomonas sp*	13	9.2
*Morganella sp*	8	5.6
*Klebsiella sp*	7	4.9
*Enterobacter sp*	7	4.9
*Escherichia Coli*	5	3.5
*Citrobacter sp*	3	2.1
*Proteus sp*	1	0.7
*Serratia sp*	1	0.7
Other gram-negative	30	21.2
Total	142	100

There were nine multidrug-resistant strains, consisting of four methicillin-resistant *Staphylococcus aureus* (MRSA), four methicillin-resistant coagulase-negative Staphylococcus (MRCoNS) and one extended-spectrum beta-lactamase (ESBL)-producing *Klebsiella sp.* In terms of antibiotic susceptibility, most of the antibiotics tested were effective against the gram-positive pathogens, except benzylpenicillin (50% sensitive), tetracyclin (50%), fusidic acid (36.36%), ciprofloxacin (0%) and cefoxitin (0%) (Table III). Similarly, gram-negative pathogens were susceptible to most of the antibiotics except cefotaxime (22.22% sensitive), amoxicillin (0%) and ampicillin-sulbactam (0%) (Table IV).

**Table III:** Antimicrobial susceptibility of gram-positive pathogens in this study

AAntibiotics	Staph. (24)	Strep. (15)	MRSA (4)	MRCONS (4)	Enterococcus (2)	Gamella (1)	Total (50)	Total (%)
Erythromycin	20/24	13/15	1/4	1/4	1/2	1/1	37/50	74
Gentamicin	22/24		3/4	0/4	0/2		25/34	73.53
Oxacillin	24/24		0/4	0/2			24/30	80
Benzylpenicillin	11/24	12/15	0/4	1/4	0/2	1/1	25/50	50
Bactrim	20/23		3/3	0/4	0/1		23/31	74.19
Fusidic acid	2/7		1/2	0/4	1/1		4/11	36.36
Clindamycin	2/5	14/15	2/4	1/4	0/1	1/1	20/30	66.67
Linezoid	1/1		3/4	4/4	2/2		10/11	90.91
Rifampicin	0/1		3/4	3/4	0/1		6/10	60
Ciprofloxacin			0/1		0/1		0/2	0
Cefoxitin			0/1	0/2	0/1		0/4	0
vancomycin			1/1	2/2	2/2		5/5	100
nitrofurantoin			1/1		1/1		2/2	100
levofloxacin			0/1		0/1		0/2	0
tetracycline			1/1		0/1		1/2	50

Bactrim – Sulfamethoxazole/trimethoprim

**Table IV:** Antimicrobial susceptibility of gram-positive pathogens in this study

Antibiotics	Pseudomonas (13)	Morganella (8)	Klebsiella (7)	Enterobacter (7)	E.Coli (5)	Citrobacter (3)	Proteus (1)	Serratia (1)	Total (45)
Ceftazidime	11/12	3/4	1/1	0/2					15/19 (78.95)
Ciprofloxacin	9/13	8/8	7/7	6/7	4/5	3/3	1/1	1/1	39/45 (86.67)
Gentamicin	10/13	7/8	7/7	6/7	5/5	3/3	1/1	1/1	40/45 (88.89)
Tazosin	10/11	4/5	5/5	6/7	5/5	3/3	1/1	1/1	35/38 (92.11)
Amikacin	2/3								2/3 (66.67)
Cefepime	2/3	4/5	7/7	6/7	5/5	3/3	1/1	1/1	29/32 (90.63)
Imipenem	1/4	0/3		2/2			0/1		3/10 (30)
Meropenem	1/3	1/1		2/2					4/6 (66.67)
Ertapenem	0/1	1/1		1/1					2/3 (66.67)
Colistin	1/1								1/1 (100)
Cefotaxime	0/4	0/1	1/1	0/2				1/1	2/9 (22.22)
Amoxicilin	0/1	0/6				0/1			0/8 (0)
Augmentin	0/1	0/4	6/7	0/7	5/5	3/3	1/1	0/1	15/29 (51.72)
Ampicillin	0/1	0/6	7/7	0/1	0/5	0/2	1/1	0/1	8/24 (33.33)
Cefuroxime	0/1	1/6	7/7	2/7	5/5	3/3	1/1	0/1	19/31 (61.29)
Bactrim		7/8	5/7	5/7	1/5	3/3	1/1	1/1	23/32 (71.88)
Unasyn		0/2							0/2 (0)

Tazosin – Piperacillin/tazobactam; Bactrim – Sulfamethoxazole/trimethoprim; Unasyn – ampicillin/sulbactam

## Discussion

Consistent with other studies in Malaysia, patients with DFI in our study show a male predominance, in which male patients outnumber female patients by more than 40%^5,17,18,20^. More than two-third of the patients do not have any identifiable precipitating factor that initiate the infection. This is probably due to diabetic neuropathy, a common microvascular complication in diabetic patients, in which they have lost the protective sensation at the foot, rendering them vulnerable to repeated microtrauma to the foot and subsequently infections^5^. This is further compounded by other complications of diabetes mellitus such as immunopathy (dysfunction immune system), vasculopathy (inadequate blood supply) and autonomic dysfunction (dry and crack skin) which contribute to diabetic foot infection^5^.

Similar to the finding of a study done at a rural area in Pahang, patients from the urban city of Pahang also tend to present late to the tertiary hospital. Kow *et al* previously demonstrated that the duration of illness is a predictive factor of major amputation in patients with DFI, hence patient education should be emphasised for patients to seek medical attention early to prevent further morbidity and mortality^17^. Most of the patients at the rural area present with infected wound which is also a finding recognised in our study at urban city^17^. The rate of major lower limb amputation for diabetic foot infection is 16.1% in this study which is lower than that reported in other countries, such as Singapore (27.2%) and Hong Kong (30.3%)^21,22^. In Malaysia, the rate of major amputation ranges from 14.2% to 20%^20,23^. A lower rate of amputation observed in Malaysia is likely due to refusal for amputation among the local population, especially major limb amputation, despite detailed counselling on its indication^24^.

In this cohort, we examine the difference between blood culture and intra-operative tissue culture in patients with DFI. In the management of sepsis, blood culture is considered the clinical gold standard to identify the causative pathogen^25^. Nevertheless, blood culture seldom shows the causative pathogen in diabetic foot infection, as evidenced by low detection rate (11.9%) among the 59 samples taken, despite all patients presenting with clinical signs of sepsis upon admission. In contrast, tissue culture is more sensitive in isolating the causative pathogens in patients with DFI as shown by 73.8% of the samples with positive yield of pathogens. Similar results are also demonstrated in the study by Son *et al*, in which 82.2% of their intra-operative tissue samples cultured pathogens^26^. With regard to the types of pathogens cultured, our finding mimics the result of Raja *et al*, another study at a teaching hospital in the urban area of Malaysia, showing a predominance of monomicrobial cultures (53.1% in this study compared to 57.2%)^18^. Along the same vein, the ratio of gram-negative to gram-positive pathogens is almost identical (with the percentage of gramnegative pathogens of 52.8%) as the one reported by Raja *et al* (52% gram-negative pathogens)^18^. This indicates that the treating physician in urban area need to take gram-positive pathogens into consideration before initiating the empirical antibiotic(s). Meanwhile, physicians can confidently initiate gram-negative-targeting antimicrobial therapy in rural areas of Malaysia, as majority of pathogens tend to be gramnegative in nature (67-95%)^5^.

Consistent with previous studies, although *Staphylococcus sp.* is the most common pathogen isolated in DFI, the percentage is much lower compared to that in western countries where it can be present in up to 50% of the diabetic foot wounds^5,16^. As for the pathogens’ antimicrobial susceptibility, gram-positive pathogens are not sensitive to penicillin (50%) and fusidic acid (36.36%). This trend is also shared among patients from rural area, revealing the possibility of resistant strains due to over-prescribed antibiotics or over-the-counter self-medication^5^. Vancomycin is the most effective antibiotic (100%), but it should be reserved to treat patients with multi-drug resistant pathogens such as methicillin-resistant *Staphylococcus aureus* (MRSA) and methicillin-resistant coagulase-negative Staphylococcus (MRCoNS). Based on the latest Malaysian Antibiotic Guidelines (2019), DFI is categorised into three groups based in its severity, namely mild, moderate and severe^27^. The preferred treatment for mild infection is oral amoxicillin/clavulanate or oral ampicillin/sulbactam. Patients with moderate infections are treated with intravenous ampicillin/sulbactam or piperacillin/tazobactam. Lastly, patients with severe infections, as evidenced by two or more systemic inflammatory response syndrome (SIRS) criteria, are treated with intravenous piperacillin/tazobactam. Microbiology profile of patients with diabetic foot infections shows a predominance of gram-negative pathogens, irrespective of urban or rural areas. They also support the usage of ampicillin/sulbactam rather than amoxicillin/ clavulanate in treatment of mild and moderate infections. The result of our study concurs with the recommendation of piperacillin-tazabactam as the most efficient (92.1%) antibiotic in treating severe infections.

Our study has several limitations. First of all, the retrospective nature of this study precludes the inclusion of several information such as HbA1c, albumin level and type of antibiotic(s) taken by patients prior to admission. In addition, our centre does not routinely perform anaerobic or fungal sampling for patients with DFI. Despite the limitations mentioned, we manage to colligate the microbiological profile of patients with DFI in this newlyestablished tertiary centre, and it can serve as a guide to dictate the type of antimicrobial therapy for patients with DFI. Nevertheless, each hospital is recommended to obtain its own microbiologic profile of patients with diabetic foot infections and treat their patients with the most appropriate empirical antibiotic(s).

## Conclusion

The microbiological profile of patients with DFI in Kuantan mimics those reported in Kuala Lumpur of Malaysia. Although Staphylococcus sp. is the most common causative pathogen, *Streptococcus sp, Pseudomonas sp,* and *Morganella sp.* are identified as significant causative organisms in our centre. This microbiological profile serves as a guide for treating physicians to initiate the most appropriate empirical antibiotic(s), based on the severity of disease.
